# Supplementary material for: Self-referential belief shares common neural correlates with general belief
Source: Sci Rep. 2025 Jan 16;15:2137. doi: 10.1038/s41598-024-84445-6 (PMC11739385; doi:10.1038/s41598-024-84445-6)
Supplement: Supplementary file 1 — Supplementary Material 1 [file 41598_2024_84445_MOESM1_ESM.docx]

**Self-Referential Belief Shares Common Neural Correlates With General Belief**

Emily Bruns, Immanuel Scholz, Georgia Koppe, Peter Kirsch, & Martin Fungisai Gerchen

**Supplementary Information**


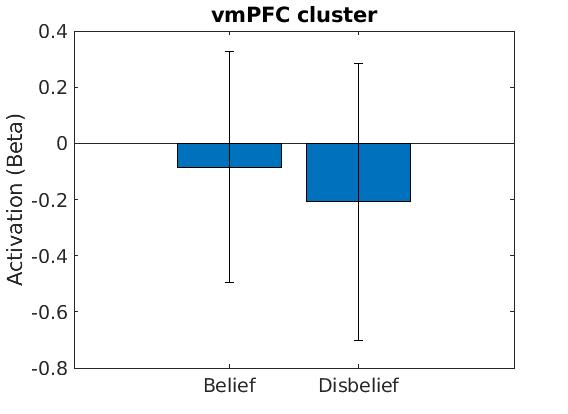


**Supplementary Figure S1. vmPFC activation.** Condition-specific activation (averaged beta values +/- 1 SD) in the vmPFC cluster in the statement phase (Figure 3a). The positive contrast effect (‘belief > disbelief’)’ is created by a stronger deactivation in the disbelief condition than in the belief condition.


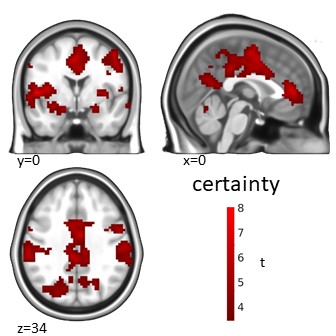


**Supplementary Figure S2. Certainty processing.** Linear relationship (parametric modulation) of activation in the statement phase with certainty over all conditions. Significance threshold p=0.05 cluster-level FWE-corrected with a cluster-defining threshold (CDT) of p=0.001 unc.

| **cluster-level** |  | **peak-level** |  |  |  |
| --- | --- | --- | --- | --- | --- |
| **p_FWE_** | **k_E_** | **p_FWE_** | **t** | **MNI**  **(mm; x, y, z)** | **label** |
| **certainty** |  |  |  |  |  |
| <0.001  <0.001  0.002  0.036  0.002 | 9845  626  129  67  122 | <0.001  0.002  0.002  0.003  0.11  0.13  0.065  0.77  0.83  0.30  0.74  0.52  0.91 | 8.03  7.69  7.65  7.41  5.85  5.73  6.10  4.58  4.49  5.29  4.62  4.92  4.32 | -9, -7, 53  -48, -4, 5  -60, -31, 20  3, 44, 8  -9, 35, -1  -9, 50, -1  30, -46, 65  30, -43, 44  24, -43, 53  24, -4, -16  12, 2, -13  66, -19, -7  63, -4, -10 | SMA  ROL  STG  ACC  ACC  ACC  postcentral gyr.  ang. gyr.  postcentral gyr.  amygdala  hippocampus  mid. temp. gyr.  mid. temp. gyr. |
|  |  |  |  |  |  |

**Supplementary Table S1. Certainty processing.** Linear relationship (parametric modulation) of activation in the statement phase with certainty over all conditions. Significance threshold p=0.05 cluster-level FWE-corrected with a cluster-defining threshold (CDT) of p=0.001 unc.


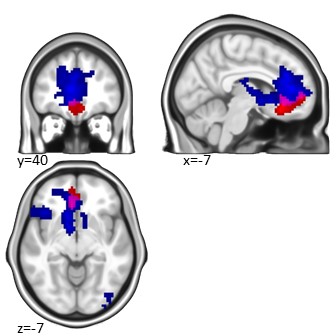


**Supplementary Figure S3. Spatial relationship of activations for self-referential processing and self-referential belief processing.** Blue: Activation for the contrast self > other (Fig. 2.). Red: Activation for the contrast self belief > self disbelief (Fig. 3b). Magenta: Overlap of the effects. While the activated clusters are contiguous and partly overlap at the border regions, under consideration of the effect of intraindividual variation and smoothing on group-level fMRI results they appear to be based on distinct neural substrates. Significance threshold p=0.05 cluster-level FWE-corrected with a cluster-defining threshold (CDT) of p=0.001 unc.


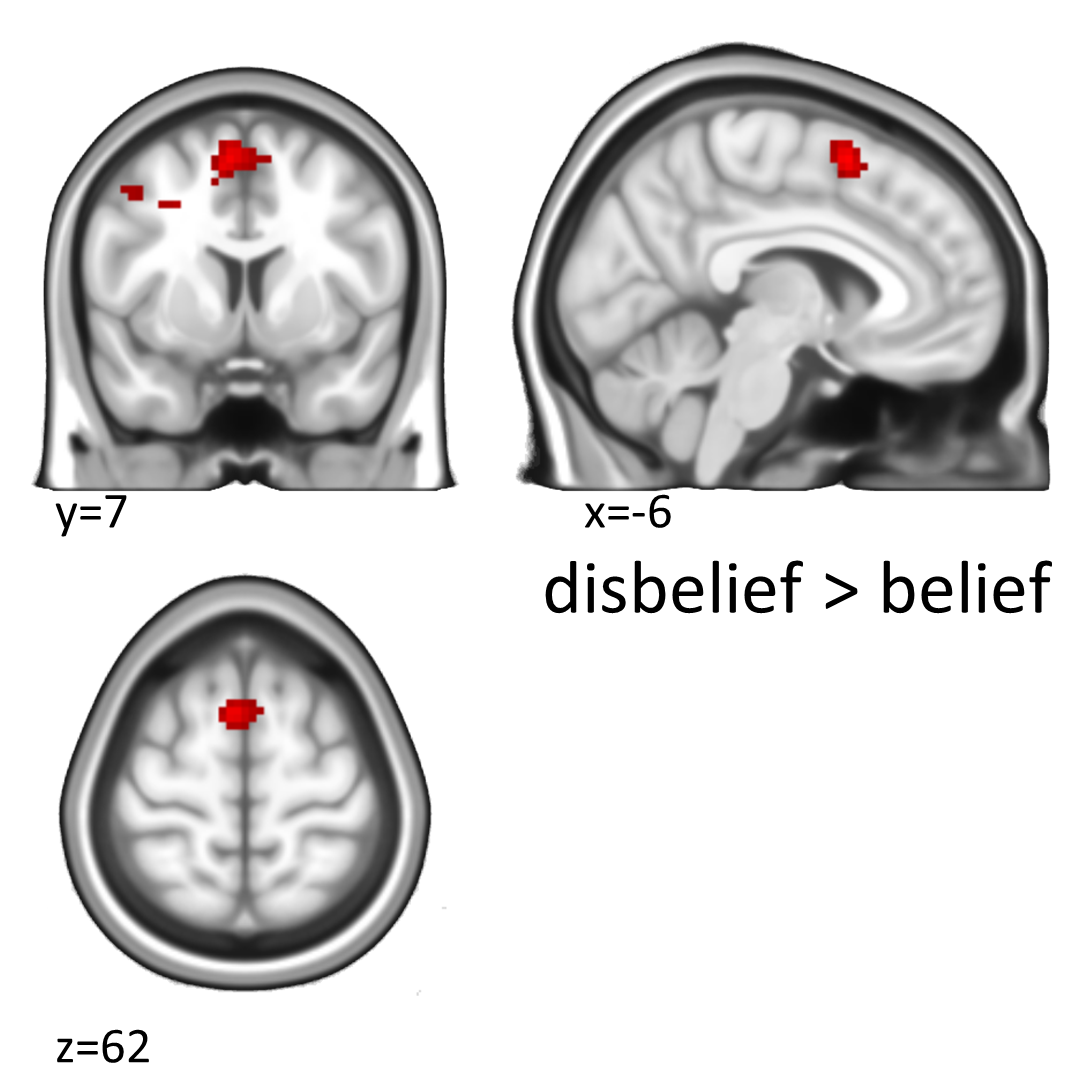


**Supplementary Figure S4. Disbelief processing in the decision phase.** Activation for the contrast ‘disbelief > belief’ in the decision phase over all conditions. Please note the similarity with the uncertainty effect in Figure 4. Significance threshold p=0.05 cluster-level FWE-corrected with a cluster-defining threshold (CDT) of p=0.001 unc.

| **Item Nr.** | **Original Item** | **English Translation** | **Set** |
| --- | --- | --- | --- |
| 1 | anpassungsfähig | adaptable | 2 |
| 2 | großzügig | generous | 1 |
| 3 | vergesslich | forgetful | 2 |
| 4 | nachdenklich | pensive | 1 |
| 5 | autoritär | authoritarian | 1 |
| 6 | intelligent | intelligent | 1 |
| 7 | leichtgläubig | gullible | 2 |
| 8 | besorgt | worried | 2 |
| 9 | empfindsam | sensitive | 2 |
| 10 | zuversichtlich | confident | 1 |
| 11 | naiv | naïve | 1 |
| 12 | skeptisch | skeptical | 1 |
| 13 | emotional | emotional | 1 |
| 14 | ehrlich | honest | 2 |
| 15 | rücksichtslos | inconsiderate | 1 |
| 16 | romantisch | romantic | 1 |
| 17 | attraktiv | attractive | 1 |
| 18 | entspannt | relaxed | 2 |
| 19 | wehleidig | whining | 1 |
| 20 | stur | stubborn | 1 |
| 21 | widerstandsfähig | robust | 2 |
| 22 | gesellig | gregarious | 1 |
| 23 | einsam | lonely | 1 |
| 24 | unberechenbar | unpredictable | 2 |
| 25 | streng | strict | 2 |
| 26 | stark | strong | 1 |
| 27 | liebenswert | kind | 2 |
| 28 | launisch | capricious | 1 |
| 29 | sarkastisch | sarcastic | 2 |
| 30 | dominant | dominant | 1 |
| 31 | taktvoll | tactful | 2 |
| 32 | kompetent | competent | 1 |
| 33 | unzufrieden | dissatisfied | 2 |
| 34 | gehemmt | inhibited | 1 |
| 35 | rational | rational | 1 |
| 36 | tolerant | tolerant | 2 |
| 37 | pessimistisch | pessimistic | 1 |
| 38 | schreckhaft | jumpy | 2 |
| 39 | bescheiden | modest | 1 |
| 40 | faul | lazy | 1 |
| 41 | normal | normal | 1 |
| 42 | provokant | provocative | 1 |
| 43 | selbstsicher | self-confident | 2 |
| 44 | loyal | loyal | 2 |
| 45 | impulsiv | impulsive | 2 |
| 46 | beharrlich | persistent | 2 |
| 47 | konsequent | consistent | 2 |
| 48 | ruhelos | restless | 1 |
| 49 | idealistisch | idealistic | 1 |
| 50 | verständnisvoll | understanding | 2 |
| 51 | liebevoll | loving | 2 |
| 52 | langweilig | boring | 1 |
| 53 | sparsam | thrifty | 2 |
| 54 | humorvoll | humorous | 2 |
| 55 | nachlässig | negligent | 2 |
| 56 | verträumt | dreamy | 1 |
| 57 | interessant | interesting | 1 |
| 58 | sprunghaft | erratic | 2 |
| 59 | sanft | gentle | 1 |
| 60 | unterhaltsam | entertaining | 2 |
| 61 | mutig | courageous | 1 |
| 62 | unflexibel | inflexible | 2 |
| 63 | unkonventionell | unconventional | 2 |
| 64 | unkompliziert | uncomplicated | 1 |
| 65 | ungeduldig | impatient | 1 |
| 66 | abenteuerlustig | adventurous | 2 |
| 67 | beliebt | popular | 1 |
| 68 | unpünktlich | unpunctual | 2 |
| 69 | stolz | proud | 2 |
| 70 | wissbegierig | inquisitive | 1 |
| 71 | berechnend | calculating | 2 |
| 72 | intellektuell | intellectual | 2 |
| 73 | gesprächig | talkative | 1 |
| 74 | ordentlich | tidy | 2 |
| 75 | ängstlich | anxious | 2 |
| 76 | ehrgeizig | ambitious | 2 |
| 77 | fleißig | hard-working | 1 |
| 78 | stabil | stable | 2 |
| 79 | unsicher | uncertain | 1 |
| 80 | materialistisch | materialistic | 2 |

**Supplementary Table S2. Study materials.** List of adjectives used in the task in original German and translated to English. ‘Set’ refers to the adjective set the word was assigned to.

|  | **Set 1 Mean ± StD** | **Set 2 Mean ± StD** | **t** | **DoF** | **p** | **Hedges’ g** |
| --- | --- | --- | --- | --- | --- | --- |
| **self-referential belief ratio** | 0.68 ± 0.07 | 0.63 ± 0.11 | 1.45 | 25 | 0.16 | 0.54 |
| **self-referential certainty** | 76.44 ± 6.53 | 78.45 ± 10.17 | -0.60 | 25 | 0.55 | -0.23 |
| **close person belief ratio** | 0.65 ± 0.08 | 0.62 ± 0.10 | 0.80 | 25 | 0.43 | 0.30 |
| **close person certainty** | 76.97 ± 6.36 | 81.59 ± 10.40 | -1.38 | 25 | 0.18 | -0.52 |
| **public person belief ratio** | 0.58 ± 0.07 | 0.49 ± 0.05 | 3.85 | 25 | <0.001 | 1.44 |
| **public person certainty** | 58.47 ± 4.93 | 65.54 ± 14.11 | -1.26 | 25 | 0.22 | -0.47 |

**Supplementary Table S3. Comparison of adjective sets.** Statistical comparison of responses (belief ratio: ratio of ‘yes’ to ‘no’ answers and certainty) in the fMRI experiment between the two adjective sets that were used in the parallel versions of the task.
